# Mapping Short Warwick and Edinburgh Mental Wellbeing Scale (SWEMWBS) to Recovering Quality of Life (ReQoL) to estimate health utilities

**DOI:** 10.1186/s12955-023-02220-z

**Published:** 2024-01-15

**Authors:** Anju Devianee Keetharuth, Laura A. Gray, Ellen McGrane, Hannah Worboys, Giovany Orozco-Leal

**Affiliations:** 1 Sheffield Centre for Health and Related Research, School of Medicine and Population Health, University of Sheffield, Sheffield, UK; 2 Department of Health Sciences, College of Life Sciences, University of Leicester, Leicester, UK; 3 Population Health Sciences Institute, Faculty of Medical Sciences, Newcastle University, Newcastle, UK

**Keywords:** Preference-based measure, Mapping, QALYs, ReQoL, SWEMWBS

## Abstract

**Background:**

The Short Warwick and Edinburgh Mental Wellbeing Scale (SWEMWBS) is a widely used non-preference-based measure of mental health in the UK. The primary aim of this paper is to construct an algorithm to translate the SWEMWBS scores to utilities using the Recovering Quality of Life Utility Index (ReQoL-UI) measure.

**Methods:**

Service users experiencing mental health difficulties were recruited in two separate cross-sectional studies in the UK. The following direct mapping functions were used: Ordinary Least Square, Tobit, Generalised Linear Models. Indirect (response) mapping was performed using seemingly unrelated ordered probit to predict responses to each of the ReQoL-UI items and subsequently to predict using UK tariffs of the ReQoL-UI from SWEMWBS. The performance of all models was assessed by the mean absolute errors, root mean square errors between the predicted and observed utilities and graphical representations across the SWEMWBS score range.

**Results:**

Analyses were based on 2573 respondents who had complete data on the ReQoL-UI items, SWEMWBS items, age and sex. The direct mapping methods predicted ReQoL-UI scores across the range of SWEMWBS scores reasonably well. Very little differences were found among the three regression specifications in terms of model fit and visual inspection when comparing modelled and actual utility values across the score range of the SWEMWBS. However, when running simulations to consider uncertainty, it is clear that response mapping is superior.

**Conclusions:**

This study presents mapping algorithms from SWEMWBS to ReQoL as an alternative way to generate utilities from SWEMWBS. The algorithm from the indirect mapping is recommended to predict utilities from the SWEMWBS.

**Supplementary Information:**

The online version contains supplementary material available at 10.1186/s12955-023-02220-z.

## Background

The constant need to improve the quality of healthcare in the NHS is reliant on the ability to assess the quality of existing and new services over time. With recent emphasis in the NHS on value-based commissioning, it is necessary to monitor and measure outcomes [1]. Quality Adjusted Life Years (QALYs) are composite measures of length of life and quality of life and provide a way of measuring the impact of the health care interventions on health-related quality of life (HRQoL). Cost per QALY is commonly used to assess the cost-effectiveness of interventions to inform resource allocation. The use of outcome measures in the United Kingdom (UK) has increased over the last decade. The Short Warwick-Edinburgh Mental Well-being Scale (SWEMWBS) is commonly used in the UK to measure mental wellbeing [2, 3]. The SWEMWBS is a validated scale capturing the positive effect of mental wellbeing. The SWEMWBS was developed from the original 14-item version, which in turn, was developed from Affectometer 2 in New Zealand and has been used with the general population, deaf people, and clinical populations including those experiencing mental health difficulties [4–8] in different settings. While a statistical relationship has been estimated between life satisfaction and SWEMWBS and is available to estimate the social value from SWEMWBS [9], it cannot be used to generate QALYs.

Utility mapping is a technique where utilities are estimated in instances when data have not been collected from preference-based measures. To develop such an algorithm, it is recommended that there is both conceptual and empirical overlap between the source measure (generally a non-preference-based measure that is being mapped from) to the target measure (generally a preference-based measure for which utilities need to be calculated) [10]. In the UK, EQ-5D is the most commonly used measure to generate QALYs in economic evaluation due to the recommendations of the National Institute for Health and Care Excellence (NICE) reference case [11]. Concerns have been raised in the literature about the validity of EQ-5D to capture health-related quality of life in the area of mental health or wellbeing [12, 13]. The focus of EQ-5D is on physical health with only one question on mental health and therefore, one can expect little conceptual overlap between EQ-5D and SWEMWBS, making EQ-5D a less suitable source measure to develop a mapping algorithm.

The Recovering Quality of Life (ReQoL) measures are validated outcome measures developed mainly for a mental health population aged 16 and over [14–16] and are being increasingly used in the UK in the general population. ReQoL-10 and ReQoL-20 comprise 10 and 20 mental health items respectively and one physical health item [17]. The first 10 items of ReQoL-20 are identical to the ReQoL-10. ReQoL-UI is the preference-based measure consisting of six mental health items and one physical health item from ReQoL-10. Preference weights for the UK were estimated from a sample of 305 from the general population using the time trade-off method [18]. Previous work has reported a large Pearson’s coefficient correlation of 0.90 between SWEMWBS and ReQoL scores [17]. Given that conceptual overlap between the two measures has been established, mapping between these two measures is a viable option. Only very recently, after the generation of our mapping algorithm, a UK preference-based value set for the SWEMWBS has been published [19]. The primary aim of this paper is to estimate an algorithm as an alternative way to predict utilities from the SWEMWBS to the ReQoL-UI. The secondary aim is to compare the different traditional mapping methods to add to the evidence base around mapping techniques.

## Methods

### Data

Data were collected from two separate studies between November 2017 and September 2018 from 18 secondary care mental health services and one general practitioner surgery across England. Participants from secondary care and primary care were recruited face-to-face (94%) and by post (6%) respectively. Participants were aged 16 and over and were mental health service users with diagnoses such as anxiety, depression, schizophrenia, other psychotic disorders (including schizo-affective disorders), bipolar disorder and personality disorder. While all participants completed SWEMWBS and demographics questions, those in Study 1 and Study 2 completed ReQoL-20 and ReQoL-10 respectively. Data were pooled to maximise sample size with a view to reducing uncertainty around estimates.

### Measures

The SWEMWBS contains seven positively worded items in which each item is answered on the following 1 to 5 frequency-based Likert scale: ‘none of the time’, ‘rarely’, ‘some of the time’, ‘often’ and ‘all of the time’. Transformed scores using Rasch analysis are recommended for the SWEMWBS, but in routine practice items are summed to produce a total score ranging from a minimum of 7 to a maximum of 35, with higher scores representing higher levels of mental wellbeing [3]. The items are around feeling optimistic about the future, feeling useful, feeling relaxed, being able to deal with problems well, thinking clearly, feeling close to other people and, being able to make up one’s own mind about things.

The ReQoL measures contains a mixture of positively and negatively worded items scored from 0 to 4 or 4 to 0 respectively where 0 represents the poorest quality of life and 4 the highest. The frequency-based response options are: ‘none of the time’, ‘only occasionally’, ‘sometimes’, ‘often’ and ‘most or all of the time’. The themes of the ReQoL measures are activity; belonging and relationships; choice; control and autonomy; hope; self-perception; wellbeing; and physical health. The ReQoL-UI is not administered as a separate measure but consists of seven items from ReQoL-10 with one item from each theme. Utilities range from − 0.195 to 1 where one represents full health and zero, the state of being dead. Values less than zero represent a perceived health state that is worse than death.

### Mapping statistical analyses

To develop mapping functions, we used both direct and indirect or response mapping. Before undertaking the mapping, it was important to determine whether to use all the SWEMWBS items or only selected ones. First, we calculated Spearman correlation where SWEMWBS items with coefficients less than 0.4 with ReQoL-UI would be considered to be weakly correlated [20]. For this study, we had decided that items with correlation coefficients of less than 0.2 would not be included unless there were deliberative reasons as to why they should be.

#### Choice of covariates

For direct mapping, the chosen SWEMWBS items were mapped to ReQoL-UI scores to capture the granularity provided by each item. The squared terms of the chosen SWEMWBS items were also included in order to capture a nonlinear relationship. For indirect mapping, we regressed each ReQoL-UI item on all the SWEMWBS items [21] and their squared terms In both types of mapping, age and sex were included as covariates as they are likely to improve the mapping functions and are usually available for participants.

#### Model types

Three model types were chosen for the direct mapping: Ordinary Least Squares (OLS), Tobit and Generalised Linear Model (GLM) (Gaussian and Gamma both with the log link). Despite its limitations, OLS remains the most used technique for mapping [22]. Therefore, ReQoL-UI was regressed on all SWEMWBS items to derive a preliminary mapping function. Given the bounded distribution of the ReQoL-UI, we also considered Tobit. However, neither of these models could take into consideration the non-normal distribution of the ReQoL-UI and therefore we estimated the GLM regressions. The GLM, an extension of OLS allows for a non-normal distribution of the dependent variable and can account for skewed and bimodal data. For the Indirect mapping, we used seemingly unrelated ordered probit and calculated the margins after each regression. We considered the significance of marginal effects [21].

#### Performance of mapping algorithms

Following the guidelines in the literature, we considered a number of measures of model fit to compare results across models [23]:mean absolute error (MAE), root mean square error (RMSE), percentage of observations with absolute errors greater than 0.1 [22], Akaike Information Criteria (AIC), Bayesian Information Criteria (BIC) and visual representation of model fit. We plotted the mean of the predicted and actual ReQoL-UI scores across the range of overall SWEMWBS scores. We also performed a simulation of patients (1000 repetitions), in order to add heterogeneity to the sample, rather than a single mean with no variation for each of the mapping models. To visually display the results of these simulations, we plotted cumulative distribution functions (CDFs). The simulations allow us to assess how well the models predict, not only at the mean (which we assess using traditional model fit statistics) but also at the extremes of the distribution. This is important for cost-effectiveness analysis when patient populations are unlikely to be the ‘average’ person and often have values that are far from the mean [24].

Throughout the study and reporting, we followed the most recent set ‘good practices’ on mapping to estimate utilities from non-preference-based measures [23]. All analyses were undertaken in STATA 17 and a mapping calculator was created in Excel 2016.

## Results

Data were collected from 2638 participants with mental health difficulties. Analyses were conducted on participants with complete data for the ReQoL-UI items, SWEMWBS items, age and sex, which led to the removal of 65 observations leaving a sample of 2573 participants. The mean (sd) age was 42 (14) years. The participants’ characteristics for the whole sample are presented in Table 1 (Table S1 presents these details for each study separately).

**Table 1:** Demographics (combined dataset - complete case *n* = 2573)

Variable		Mean (SD)	Range
Age		42 (14)	(17, 89)
Life Satisfaction		4.71 (2.83)	(1, 10)
		N	%
Sex	Male	1245	48.39
	Female	1328	51.61
Ethnicity	White	2030	78.90
	Non-White	495	19.24
	Missing	48	1.87
Diagnosis^a^	Depression	1021	39.68
	Anxiety	793	30.82
	Schizophrenia and other psychotic disorders	620	24.10
	Bipolar	409	15.90
	Personality disorders	294	11.43
Education (attended school till minimum age)	Yes	1657	64.40
	No	903	35.10
	Missing	13	0.51
Degree	Yes	838	32.57
	No	1707	66.34
	Missing	28	1.09
Main Activity	Employed	693	26.93
	Unemployed	1848	71.82
	Missing	32	1.24
General Health	Excellent	169	6.57
	Very Good	388	15.08
	Good	712	27.67
	Fair	736	28.60
	Poor	560	21.76
	Missing	8	0.31
General Mental Health	Very Poor	372	14.46
	Poor	744	28.92
	Fair	735	28.57
	Good	514	19.98
	Excellent	191	17.42
	Missing	17	0.66

^a^The numbers and percentages reflect the fact that participants had several mental health conditions

**Fig. 1:**
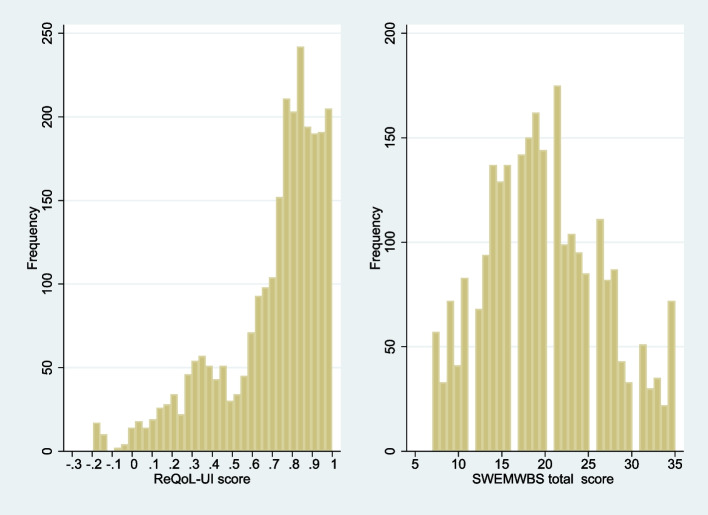
Distribution of ReQoL-UI and SWEMWBS scores. **a** Distribution of ReQoL-UI score, **b** Distribution of SWEMWBS total score

Both ReQoL and SWEMWBS scores spanned the entire range of possible values (Table 2). We have included the seven ReQoL items that are used to calculate the ReQoL-UI. Figure 1 shows the distributions of ReQoL-UI and SWEMWBS. The ReQoL-UI distribution is not normally distributed but instead, it is multimodal with a spike at full health. The SWEMWBS distribution is more normally distributed but, with gaps at some scores. For the ReQoL-UI, there are 64 (2.5%) and 41 (1.6%) observations at the best and worst health state respectively. For the SWEMWBS, there are 72 (2.8%) and 57 (2.2%) observations at the highest and lowest possible scores respectively. The frequency endorsement for ReQoL-UI and SWEMWBS are presented in Tables S2a-b (Supplementary materials).

**Table 2:** Distribution of scores and responses by source and target measures

(*n* = 2573)	Mean ± sd	Minimum	Maximum
**ReQoL-UI score**	0.698 ± 0.258	− 0.195	1
**ReQoL items**			
ReQoL 3. I felt unable to cope	2.035 ± 1.362	0	4
ReQoL 5. I felt happy	1.775 ± 1.279	0	4
ReQoL 6. I thought my life was not worth living	2.530 ± 1.462	0	4
ReQoL 7. I enjoyed what I did	1.964 ± 1.269	0	4
ReQoL 9. I felt lonely	1.892 ± 1.431	0	4
ReQoL 10. I felt confident in myself	1.611 ± 1.356	0	4
ReQoL physical health question	2.562 ± 1.237	0	4
**SWEMWBS score**	19.890 ± 6.764	7	35
**SWEMWBS items**			
1. I’ve been feeling optimistic about the future	2.716 ± 1.246	1	5
2. I’ve been feeling useful	2.717 ± 1.203	1	5
3. I’ve been feeling relaxed	2.652 ± 1.160	1	5
4. I’ve been dealing with problems well	2.827 ± 1.200	1	5
5. I’ve been thinking clearly	2.917 ± 1.202	1	5
6. I’ve been feeling close to other people	2.821 ± 1.241	1	5
7. I’ve been able to make my own minds about things	3.241 ± 1.188	1	5

### Correlation of items

The Spearman rank correlation between ReQoL-UI and each SWEMWBS item ranged between 0.498 and 0.599, which indicated that better predictions would be obtained if all items were used. The correlation between SWEMWBS score and the ReQoL-UI score was 0.593 (Table S3-S4 Supplementary materials). The correlations between SWEMWBS items and ReQoL mental health items ranged from 0.382 to 0.607 with the smallest correlations observed between SWEMWBS items and the physical item in ReQoL-UI with correlation coefficients ranging from 0.204 to 0.266. Therefore, in the mapping models, all SWEMWBS items were included in the regression.

### Model performance

The results by model type are presented in Table 3 below.

**Table 3:** Comparison of model fits

	OLS	Tobit	GLM Gaussian log link	Response Mapping
Mean Absolute Error (MAE)	0.147	0.147	0.149	0.156
Root Mean Square Error (RMSE)	0.197	0.197	0.198	0.199
Number of observations with Absolute Error (AE) > 0.05	1919	1892	1951	2141
Percentage of observations with AE > 0.05	75%	74%	76%	83%
Number of observations with AE > 0.1	1371	1361	1403	1545
Percentage of observations with AE > 0.1	53%	53%	55%	60%
Akaike Information Criteria (AIC)	− 1027	− 817	− 1003	N/A
Bayesian Information Criteria (BIC)	− 927	− 711	− 903	N/A

AIC and BIC are not included for response mapping because they are not comparable with the others

### Direct mapping

**Fig. 2:**
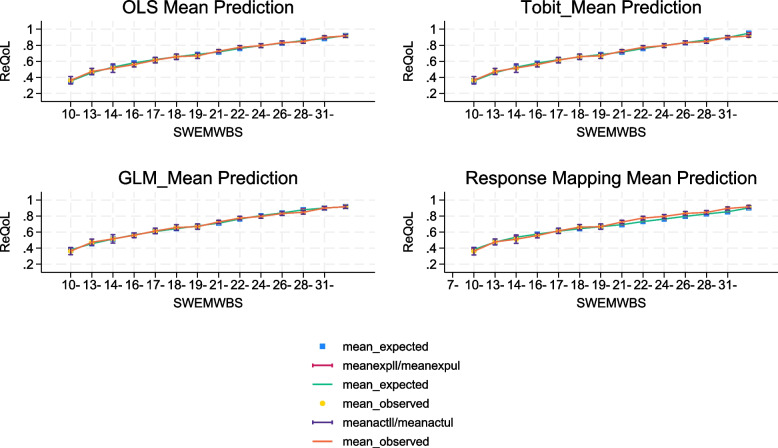
Predicted versus actual utilities by SWEMWBS score. **a** Predicted versus actual utilities (OLS), **b** Predicted versus actual utilities (Tobit), **c** Predicted versus actual utilities (GLM), **d** Predicted versus actual utilities (response mapping)

**Fig. 3:**
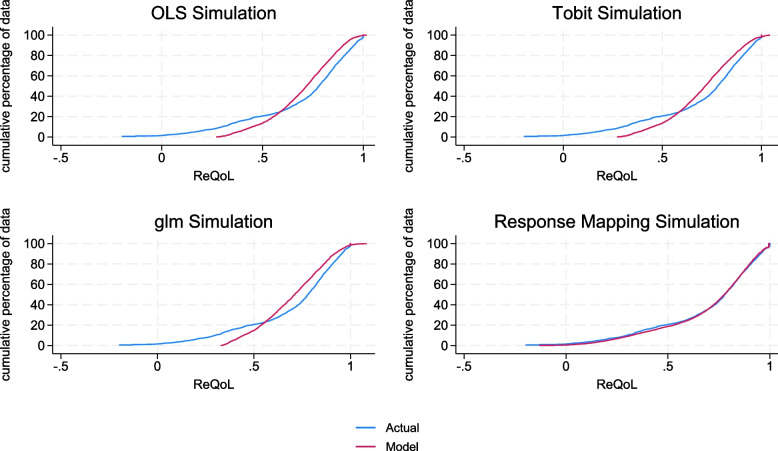
Cumulative distribution functions from simulations. **a** OLS regression model, **b** Tobit regression model, **c** GLM regression model and **d** Response mapping model

The model fits for all the three models were very similar. MAE (RMSE) were 0.147 (0.197) for both OLS models and Tobit models. MAE (RMSE) were 0.149 (0.198) for the GLM specification. The number of observations with absolute error (AE) greater than 0.05 ranged from 53 to 55%. From the graphical representations (Fig. 2), there is no systematic pattern of predictions over and below the observed values by SWEMWBS scores. However, the results from the simulations, which present the model performance across the spectrum of utility (Fig. 3), show that the direct mapping methods has a clear disparity between the observed and predicted data across the entire distribution of SWEMWBS. The GLM models with the Gaussian log link had lower AIC and BIC compared with the Gamma log link, therefore the Gamma log link results are not presented in this paper. The regression coefficients generated from the three model specifications can be found in Table S6 in the Supplementary Materials.

### Indirect mapping

The MAE and RMSE for the response mapping were 0.156 and 0.199 respectively, marginally higher than the errors produced using the direct mapping methods. However, Fig. 3 shows that there is much less bias, regardless of ReQoL status when using the response mapping, which fits the data very closely across all SWEMWBS scores.

## Discussion

This study aimed to develop a mapping algorithm to predict ReQoL-UI scores from the widely used SWEMWBS. We have mapped SWEMWBS to ReQoL using different regression techniques from the simplest one to more sophisticated ones. Given the previous inability to calculate utilities from the SWEMWBS, the mapping algorithms developed will enable researchers to produce utilities from the ReQoL-UI. We have considered not only the model fit for the means of the distribution, but also used simulated data to consider heterogeneity making the mapping algorithm more appropriate for use in cost-utility studies. The detailed results are presented in the Supplementary materials of this paper. An algorithm for the response mapping has been estimated to generate the ReQoL-UI scores and is available in Excel in the Supplementary materials.

Physical health was identified as an important theme in the life of people with mental health conditions in the early development of the ReQoL [17, 18, 25]. This theme is not captured by the SWEMWBS, hence the weak correlations observed between the SWEMWBS items and the physical item of the ReQoL-UI. While this is likely to make predictions less accurate, until preference weights are elicited for the SWEMWBS, ReQoL-UI remains the most appropriate measure to generate utilities from SWEMWBS given that both measures capture mental wellbeing.

For the direct mapping methods, we found very little differences among the three regression specifications used in terms of model fit and visual inspection of modelled and actual utility values across the SWEMWBS score range. The response mapping showed the highest proportion (60%) of observations with AE > 0.05. However, the comparison of mapping techniques and model specifications in this paper illustrates the importance of looking at uncertainty around model predictions and the model outputs once patient variability is considered. All models estimated mean utility well, including when looking specifically at observations grouped by total SWEMWBS score. However, using simulated data, we showed that response mapping outperformed the other mapping techniques once patient variability was taken into account. This is particularly important if the mapping algorithm is to be used for cost-utility analysis. The mean errors do not always give a good representation of model fit if the majority of observations are at the same part of a distribution where a model fits well. Observations are more difficult to estimate for parts of the distribution (for example at the severe end of utilities) and may be under represented in the data, but it is important that they are also estimated accurately for cost-effectiveness analysis, in line with findings from other papers [24, 26–29]. Therefore, we recommend the response mapping to generate ReQoL-UI scores from SWEMWBS responses if estimates are going to be used for economic evaluation.

The algorithms presented here are also a useful way of comparing SWEMWBS scores and scores from ReQoL-10 and ReQoL-20. In the UK, mental health trusts and other charities have either used one of the ReQoL measures or SWEMWBS. There may be reasons to compare the SWEMBWS and ReQoL scores when only one of the measures has been administered. For this purpose, ideally, we would produce separate mapping functions between the two measures because the correlation between SWEWMBS and ReQoL-10 is higher than with ReQoL-UI. This difference can be accounted for by the fact that the ReQoL scores do not include the physical item while ReQoL-UI does. However, in the absence of mapping functions between SWEMWBS and ReQoL-10 and ReQoL-20, the algorithms presented here can be used to compare the two measures.

This study has several limitations. First, the mapping was performed using data from a population experiencing a broad range of mental health difficulties. The mapping functions need to be tested in other populations to assess where their use could be extended to the general population and other populations. Second, it is recognised that, while the algorithm is recommended for use for populations similar to the ones in this study, it may not be applicable in very different populations. Third, we have not explored more recently developed mapping techniques like the use of mixture models. There is some evidence that mixture models can produce more accurate predictions because they better estimate the unusual, non-normal and limited distributions common among health utility data [24]. However, future research is needed into how mixture models predict ReQoL utilities.

In this case, by using indirect mapping, we can overcome some of the problems associated with more commonly used traditional mapping methods. Using OLS can lead to predictions outside the feasible range of utility values. The Tobit model can handle the limited nature of preference-based measures by limiting predicted values at 1 (full health). The GLM models are limited as they are unable to predict negative values. The OLS, Tobit and GLM models also fail to capture the multimodal nature of ReQoL. The indirect mapping method used in this study allows for a more flexible approach whilst also predicting values within the feasible range, by estimating the probabilities of each ReQoL dimension score, then calculating the expected ReQoL utility value by the weighted probabilities.

## Conclusions

This is the first study to map from SWEMWBS to any preference-based measure. The paper presents mapping functions to generate utility values from SWEMWBS to ReQoL-UI. When only point estimates are considered, there is little difference between the various mapping methods. However, when heterogeneity is considered, response mapping outperforms the direct mapping methods. The use of the algorithm using the indirect mapping technique is therefore recommended to generate utilities for use in cost-utility analyses. We have produced a tool in the form of a calculator to help research to easily compute utilities from SWEMWBS. Future research is needed to compare the values generated from the mapping algorithm with those directly generated from the new set of preference weights elicited using health states from the SWEMWBS.

### Supplementary Information

**Additional file 1.**

**Additional file 2.**

## Data Availability

The data that support the findings of this study are available from the corresponding author upon reasonable request.

## Abbreviations

AE: Absolute error
AIC: Akaike information criteria
BIC: Bayesian information criteria
GLM: Generalised linear model
HRQoL: Health-related quality of life
MAE: Mean absolute error
NICE: National institute for health and care excellence
OLS: Ordinary least squares
QALYs: Quality adjusted life years
ReQoL: Recovering Quality of Life
ReQoL-UI: Recovering Quality of Life - Utility Index
RMSE: Root mean square error
SWEMWBS: Short Warwick-Edinburgh Mental Well-being Scale
UK: United Kingdom
