# Supplementary material for: Mapping Short Warwick and Edinburgh Mental Wellbeing Scale (SWEMWBS) to Recovering Quality of Life (ReQoL) to estimate health utilities
Source: Health Qual Life Outcomes. 2024 Jan 15;22:7. doi: 10.1186/s12955-023-02220-z (PMC10789009; doi:10.1186/s12955-023-02220-z)
Supplement: Supplementary file 1 — Additional file 1. [file 12955_2023_2220_MOESM1_ESM.docx]

**Tables and figures - supplementary materials**

Table S1 Participants’ characteristics by study

|  |  | Study 1  n = 1984 | | Study 2  n = 589 | |  |
| --- | --- | --- | --- | --- | --- | --- |
| *Variable* |  | *Mean (SD)* | *Range* | *Mean (SD)* | *Range* | *p value* |
| Age |  | 42 (14) | 17 to 89 | 42 (14) | 18 to 81 | 0.26 |
| Life Satisfaction |  | 4.76 (2.80) | 0 to 10 | 4.55 (2.94) | 0 to 10 | 0.12 |
|  |  | N | % | N | % |  |
| Sex | Male | 946 | 47.68 | 299 | 50.76 | 0.19 |
|  | Female | 1038 | 52.32 | 290 | 49.24 |  |
| Ethnicity | White | 1541 | 77.67 | 489 | 83.02 | 0.01*** |
|  | Non-White | 403 | 20.31 | 92 | 15.62 |  |
|  | Missing | 40 | 2.02 | 8 | 1.36 |  |
| Diagnosis^a^ | Depression | 777 | 39.16 | 244 | 41.43 | 0.32 |
|  | Anxiety | 612 | 30.85 | 181 | 30.73 | 0.96 |
|  | Schizophrenia and other psychotic disorders | 454 | 22.88 | 166 | 28.18 | 0.01*** |
|  | Bipolar | 340 | 17.14 | 69 | 11.71 | 0.02*** |
|  | Personality disorder | 238 | 12.00 | 56 | 9.51 | 0.10* |
| Education (attended school till minimum age) | Yes | 1295 | 65.27 | 362 | 61.46 | 0.07* |
|  | No | 677 | 34.12 | 226 | 38.37 |  |
|  | Missing | 12 | 0.60 | 1 | 0.17 |  |
| Degree | Yes | 656 | 33.06 | 182 | 30.90 | 0.29 |
|  | No | 1304 | 65.73 | 403 | 68.42 |  |
|  | Missing | 24 | 1.21 | 4 | 0.68 |  |
| Main Activity | Employed | 539 | 27.17 | 154 | 26.15 | 0.58 |
|  | Unemployed | 1418 | 71.47 | 430 | 73.01 |  |
|  | Missing | 27 | 1.36 | 5 | 0.85 |  |
| General Health | Excellent | 129 | 6.50 | 40 | 6.79 | 0.87 |
|  | Very Good | 292 | 14.72 | 96 | 16.30 |  |
|  | Good | 563 | 28.38 | 149 | 25.30 |  |
|  | Fair | 569 | 28.68 | 167 | 28.35 |  |
|  | Poor | 424 | 21.37 | 136 | 23.09 |  |
|  | Missing | 7 | 0.35 | 1 | 0.17 |  |
| General Mental Health | Very Poor | 266 | 13.41 | 106 | 18.00 | 0.19 |
|  | Poor | 582 | 29.33 | 162 | 27.50 |  |
|  | Fair | 582 | 29.33 | 153 | 25.98 |  |
|  | Good | 390 | 19.66 | 124 | 21.05 |  |
|  | Excellent | 150 | 7.56 | 41 | 6.96 |  |
|  | Missing | 14 | 0.71 | 3 | 0.51 |  |

^a^ The numbers and percentages reflect the fact that participants had several mental health conditions

Table S2a Frequency endorsement for ReQoL-UI items

|  | ReQoL 3 | ReQoL 5 | ReQoL 6 | ReQoL 7 | ReQoL 9 | ReQoL 10 | ReQoL physical |
| --- | --- | --- | --- | --- | --- | --- | --- |
| Level 0 | 441 | 455 | 371 | 366 | 585 | 692 | 161 |
| Level 1 | 507 | 723 | 324 | 598 | 517 | 623 | 390 |
| Level 2 | 659 | 710 | 441 | 795 | 576 | 612 | 643 |
| Level 3 | 454 | 315 | 444 | 390 | 380 | 286 | 601 |
| Level 4 | 512 | 370 | 993 | 424 | 515 | 360 | 778 |
| Total | 2573 | 2573 | 2573 | 2573 | 2573 | 2573 | 2573 |

Table S2b Frequency endorsement for SWEMWBS items

|  | SWEMWBS 1 | SWEMWBS 2 | SWEMWBS 3 | SWEMWBS 4 | SWEMWBS 5 | SWEMWBS 6 | SWEMWBS 7 |
| --- | --- | --- | --- | --- | --- | --- | --- |
| None of the time | 514 | 463 | 458 | 423 | 360 | 452 | 230 |
| Rarely | 639 | 688 | 753 | 579 | 582 | 602 | 434 |
| Some of the time | 771 | 788 | 795 | 861 | 852 | 768 | 853 |
| Often | 363 | 383 | 361 | 442 | 468 | 456 | 599 |
| All of the time | 286 | 251 | 206 | 268 | 311 | 295 | 457 |
| Total | 2573 | 2573 | 2573 | 2573 | 2573 | 2573 | 2573 |

Table S3 Pearson correlation of SWEMWBS with ReQoL-10 scores and ReQoL-UI scores

|  | Correlation coefficient |
| --- | --- |
| ReQoL-10 score and SWEMWBS score | 0.833 |
| ReQoL-UI score and SWEMWBS score | 0.593 |
| ReQoL-10 and ReQoL-UI | 0.704 |

Table S4 Spearman correlation of SWEMWBS with ReQoL-UI items

|  | | | | | | | | |
| --- | --- | --- | --- | --- | --- | --- | --- | --- |
|  | ReQoL 3 | ReQoL 5 | ReQoL 6 | ReQoL 7 | ReQoL 9 | ReQoL 10 | ReQoL physical | Utility score |
| SWEMWBS 1 | 0.4377 | 0.554 | 0.4909 | 0.382 | 0.382 | 0.6069 | 0.2045 | 0.5524 |
| SWEMWBS 2 | 0.4571 | 0.5615 | 0.4882 | 0.4065 | 0.4065 | 0.5946 | 0.2171 | 0.5617 |
| SWEMWBS 3 | 0.4778 | 0.5925 | 0.4825 | 0.4342 | 0.4342 | 0.5908 | 0.2641 | 0.5985 |
| SWEMWBS 4 | 0.504 | 0.5452 | 0.5027 | 0.4386 | 0.4386 | 0.6088 | 0.255 | 0.5961 |
| SWEMWBS 5 | 0.5098 | 0.554 | 0.5249 | 0.4323 | 0.4323 | 0.6008 | 0.2661 | 0.599 |
| SWEMWBS 6 | 0.3514 | 0.5222 | 0.434 | 0.4167 | 0.4167 | 0.4917 | 0.204 | 0.498 |
| SWEMWBS 7 | 0.4316 | 0.4762 | 0.4428 | 0.3739 | 0.3739 | 0.5273 | 0.2268 | 0.519 |

Table S5 Results from oprobit models

|  | (1) | (2) | (3) | (4) | (5) | (6) | (7) |
| --- | --- | --- | --- | --- | --- | --- | --- |
|  | ReQoL 3 | ReQoL 5 | ReQoL 6 | ReQoL 7 | ReQoL 9 | ReQoL 10 | ReQoL physical |
| 1.wem1 | 0 | 0 | 0 | 0 | 0 | 0 | 0 |
| 2.wem1 | 0.163* | 0.177* | 0.280*** | 0.150* | -0.0496 | 0.256** | 0.111 |
| 3.wem1 | 0.296*** | 0.477*** | 0.541*** | 0.340*** | 0.109 | 0.588*** | 0.0712 |
| 4.wem1 | 0.315** | 0.608*** | 0.657*** | 0.446*** | -0.00480 | 0.822*** | 0.0586 |
| 5.wem1 | 0.243* | 0.504*** | 0.435*** | 0.436*** | 0.115 | 1.039*** | -0.0297 |
| 1.wem2 | 0 | 0 | 0 | 0 | 0 | 0 | 0 |
| 2.wem2 | 0.144 | 0.215** | 0.165* | 0.288*** | 0.0520 | 0.293*** | -0.00209 |
| 3.wem2 | 0.329*** | 0.463*** | 0.350*** | 0.522*** | 0.212** | 0.422*** | 0.0497 |
| 4.wem2 | 0.387*** | 0.556*** | 0.330** | 0.720*** | 0.239* | 0.694*** | -0.00571 |
| 5.wem2 | 0.323* | 0.577*** | 0.533*** | 0.626*** | 0.216 | 0.700*** | 0.0736 |
| 1.wem3 | 0 | 0 | 0 | 0 | 0 | 0 | 0 |
| 2.wem3 | 0.0635 | 0.341*** | 0.0257 | 0.179* | 0.163* | 0.112 | 0.0576 |
| 3.wem3 | 0.262** | 0.534*** | 0.188* | 0.485*** | 0.300*** | 0.316*** | 0.196* |
| 4.wem3 | 0.496*** | 0.935*** | 0.323** | 0.756*** | 0.439*** | 0.499*** | 0.292** |
| 5.wem3 | 0.468*** | 1.311*** | 0.362* | 1.223*** | 0.750*** | 0.992*** | 0.567*** |
| 1.wem4 | 0 | 0 | 0 | 0 | 0 | 0 | 0 |
| 2.wem4 | 0.174* | 0.194* | 0.171* | 0.0976 | 0.173* | 0.315*** | -0.0221 |
| 3.wem4 | 0.409*** | 0.254** | 0.331*** | 0.145 | 0.360*** | 0.470*** | 0.0951 |
| 4.wem4 | 0.547*** | 0.389*** | 0.424*** | 0.183 | 0.486*** | 0.631*** | 0.198 |
| 5.wem4 | 0.706*** | 0.263* | 0.416** | 0.291* | 0.432*** | 0.903*** | 0.217 |
| 1.wem5 | 0 | 0 | 0 | 0 | 0 | 0 | 0 |
| 2.wem5 | 0.0208 | 0.00116 | -0.00936 | -0.00481 | -0.0836 | -0.0531 | -0.00862 |
| 3.wem5 | 0.209* | 0.0710 | 0.250** | 0.0748 | -0.0230 | 0.169 | 0.0671 |
| 4.wem5 | 0.483*** | 0.233* | 0.462*** | 0.177 | 0.0952 | 0.262* | 0.192 |
| 5.wem5 | 0.659*** | 0.303* | 0.713*** | 0.130 | 0.343* | 0.560*** | 0.272* |
| 1.wem6 | 0 | 0 | 0 | 0 | 0 | 0 | 0 |
| 2.wem6 | -0.0612 | 0.243** | 0.111 | 0.186* | 0.191* | 0.112 | 0.203** |
| 3.wem6 | -0.0970 | 0.354*** | 0.252*** | 0.243*** | 0.322*** | 0.175* | 0.170* |
| 4.wem6 | -0.0442 | 0.688*** | 0.360*** | 0.544*** | 0.604*** | 0.315*** | 0.150 |
| 5.wem6 | -0.145 | 0.702*** | 0.316** | 0.647*** | 0.559*** | 0.137 | 0.130 |
| 1.wem7 | 0 | 0 | 0 | 0 | 0 | 0 | 0 |
| 2.wem7 | -0.153 | -0.186 | -0.112 | -0.0165 | -0.129 | -0.125 | 0.129 |
| 3.wem7 | -0.0635 | -0.125 | -0.105 | 0.0531 | -0.137 | -0.0798 | 0.0279 |
| 4.wem7 | 0.111 | -0.190 | 0.0564 | 0.153 | -0.0294 | 0.0491 | 0.282** |
| 5.wem7 | 0.220 | 0.195 | 0.246* | 0.411*** | 0.0453 | 0.360** | 0.108 |
| cut1 | -0.0314 | 0.428*** | 0.0687 | 0.193* | 0.0817 | 0.906*** | -0.967*** |
| cut2 | 0.725*** | 1.586*** | 0.661*** | 1.185*** | 0.764*** | 1.876*** | -0.180* |
| cut3 | 1.563*** | 2.686*** | 1.277*** | 2.334*** | 1.463*** | 2.911*** | 0.572*** |
| cut4 | 2.248*** | 3.345*** | 1.862*** | 3.063*** | 2.009*** | 3.598*** | 1.217*** |
| N | 2573 | 2573 | 2573 | 2573 | 2573 | 2573 | 2573 |

** p<0.01 *** p<0.001

Table S6 Regression coefficients from direct mapping

| Covariates | OLS | Tobit | GLM  Gamma log | GLM  Gaussian log |
| --- | --- | --- | --- | --- |
| SWEMWBS 1 | 0.103***  (5.64) | 0.100*** (5.41) | 0.190*** (5.57) | 0.144*** (5.20) |
| SWEMWBS 2 | 0.0738***  (3.85) | 0.0712***  (3.66) | 0.120*** (3.34) | 0.126*** (4.31) |
| SWEMWBS 3 | 0.0360  (1.90) | 0.0271  (1.41) | 0.0909** (2.62) | 0.0714* (2.41) |
| SWEMWBS 4 | 0.0782***  (3.94) | 0.0738***  (3.66) | 0.143*** (3.83) | 0.141*** (4.56) |
| SWEMWBS 5 | 0.0322  (1.55) | 0.0287 (1.36) | 0.0731 (1.88) | 0.0883** (2.64) |
| SWEMWBS 6 | 0.0564***  (3.33) | 0.0529** (3.08) | 0.105*** (3.32) | 0.0783** (3.02) |
| SWEMWBS 7 | -0.000510  (-0.03) | 0.00118 (0.06) | 0.00331 (0.09) | 0.0319 (1.00) |
| SWEMWBS 1^2^ | -0.0147***  (-4.73) | -0.0143*** (-4.52) | -0.0279*** (-4.78) | -0.0203*** (-4.52) |
| SWEMWBS 2^2^ | -0.00958**  (-2.90) | -0.00895** (-2.67) | -0.0154* (-2.49) | -0.0166*** (-3.51) |
| SWEMWBS 3^2^ | 0.00108  (0.33) | 0.00310 (0.92) | -0.00453 (-0.75) | -0.00272 (-0.57) |
| SWEMWBS 4^2^ | -0.00842*  (-2.51) | -0.00744* (-2.18) | -0.0168** (-2.66) | -0.0162*** (-3.32) |
| SWEMWBS 5^2^ | -0.000828  (-0.24) | -0.0000446 (-0.01) | -0.00499 (-0.77) | -0.00777 (-1.52) |
| SWEMWBS 6^2^ | -0.00710*  (-2.48) | -0.00634* (-2.18) | -0.0144** (-2.67) | -0.00948* (-2.29) |
| SWEMWBS 7^2^ | 0.00241  (0.79) | 0.00218 (0.71) | 0.00344 (0.60) | -0.00133 (-0.28) |
| age | -0.00248***  (-8.62) | -0.00256*** (-8.76) | -0.00402*** (-7.49) | -0.00322*** (-7.99) |
| female | -0.00725  (-0.92) | -0.00679 (-0.84) | -0.00578 (-0.39) | -0.0124 (-1.11) |
| _cons | 0.0914**  (2.84) | 0.116*** (3.54) | -1.499*** (-24.87) | -1.462*** (-25.66) |
| var(e.util~) |  | 0.0400*** (35.35) |  |  |
| AIC |  |  | 3160.793 | -1003.259 |
| BIC |  |  | 3260.291 | -903.760 |

t statistics in parentheses * p<0.05, ** p<0.01, *** p<0.001

Figure S1 Score distributions ReQoL, SWEMWBS (histogram)
